# Case of Phrenitic Inflammation, in Which Benefit (by Metastasis?) Was Derived from Croton Oil

**Published:** 1827-07

**Authors:** John Frost


### *Case of Phrenitic Inflammation, in which benefit (by Metastasis?) was derived from Croton Oil.* By John Frost, F.A.S. F.L.S. &c. &c.

Knowing the interest which you take in promoting medical science, I am induced to communicate, through the medium of your Journal, the following remarks. I am convinced of the importance of this subject, in which I have been engaged for several years. The subjoined case is one amongst many others wherein I have observed that the peculiar action of the expressed oil of the seed of Croton Tiglium on the throat and fauces, has been attended with the best effects.

A robust, plethoric gentleman was seized, about twelve days ago, with an attack of phrenitis, to relieve which the usual antiphlogistic method of treatment was adopted to the fullest extent: copious bleeding to the amount of fifty ounces,—active cathartics of scammony, gamboge, and calomel in large doses,—together with the application of a solution of muriate of ammonia and nitrate of potassa to the head, were persevered in, without any diminution in the urgency of the symptoms; the pulse continuing upwards of 120 in a minute. An unfavourable prognosis only could be formed under such circumstances.

I administered one drop of the oil of Croton Tiglium-seed,* which in ten minutes produced violent hypercatharsis, and in the course of eight hours the patient became gradually sensible, although furious mania had existed, without a moment's intermission, for upwards of two days. On recovering his reason, he complained of very great difficulty of deglutition, which was evidently caused (on inspecting the throat and tonsils) by the croton oil, which certainly produces an active inflammation *sui generis*. By the use of demulcents, this increased action was subdued, and there was no return of the former symptoms. It is remarkable

* As much difference of opinion exists as to the activity of the Croton Tiglium-seed oil generally sold, I think it right to say that I purchased that above referred to of Mr. Short, of Ratcliff Highway, whose son-in-law, Dr. Conwell, revived its use in this country in 1819.

that, on pouring out some of the oil in question, a drop happened to fall on one of the fingers of my right hand, which caused pain in the throat, together with a feeling of heat, which lasted for two or three hours.

From the foregoing case, it would appear that a metastasis of inflammation from the brain to the throat was speedily effected; and, as the latter inflammation was artificial, it subsided when the stimulus was withdrawn.

I would here suggest an idea which may, perhaps, prove useful: it is that of besmearing the tonsils of a person labouring under hydrophobia with a portion of this oil. Might not the symptoms be alleviated? and would not the spasmodic action be lessened, and the pharyngeal inflammation altered, by producing one of a different and peculiar kind? At any rate, there is a possibility of advantage; and, as the experiment has never been tried, this suggestion may be useful.

The active principle of the expressed croton oil is entirely soluble in ether and oil of turpentine, and partially so in alcohol and proof spirit. I hope shortly to lay before the public some experiments, which will prove the existence of this peculiar principle in some other genera of plants, which I trust may not be unworthy the attention of the medical profession.

*London; 18th May,* 1827.

